# STX2 Promotes Trophoblast Growth, Migration, and Invasion Through Activation of the PI3K-AKT Pathway in Preeclampsia

**DOI:** 10.3389/fcell.2021.615973

**Published:** 2021-07-06

**Authors:** Yan Li, Xian-li Sun, Chun-ling Ma, Chao Li, Ying Zhan, Wen-ting Li, Can Li, Yi-hao Wang

**Affiliations:** ^1^Department of Obstetrics and Gynaecology, The Affiliated Hospital of Qingdao University, Qingdao University, Qingdao, China; ^2^Department of Obstetrics and Gynecology, Qingdao Women and Children’s Hospital, Qingdao University, Qingdao, China; ^3^Department of Pain Management, The Affiliated Hospital of Qingdao University, Qingdao University, Qingdao, China

**Keywords:** syntaxin2, trophoblast, PI3K, AKT, preeclampsia

## Abstract

**Objectives:**

Abnormal trophoblast behaviors during pregnancy contribute to the development of preeclampsia (PE). Syntaxin2 (STX2) has been shown to be a crucial epithelial mediator in numerous diseases. However, the functions of STX2 and the mechanisms underlying its role in PE remain largely unknown. The aim of this study was to explore the role of STX2 on trophoblast biology and unravel the molecular mechanisms that contribute to the development and progression of PE.

**Materials and Methods:**

We first compared the expression of STX2 in placental tissues from women with PE and women with normal pregnancies. Then, we investigated the role of STX2 on trophoblast proliferation, migration and invasion in HTR-8/SVneo and primary human trophoblast cells by loss or gain of function experiments. In addition, co-immunoprecipitation, pulldown and immunofluorescence assays were performed to investigate the co-localization of STX2 with other proteins, and to help clarify the mechanisms underlying STX2-mediated functions on trophoblasts.

**Results:**

We demonstrated that STX2 expression was downregulated in placental tissues of women with PE compared with those from normal pregnancies. Loss and gain of function experiments further confirmed a role for STX2 in cell proliferation, migration and invasion in trophoblasts. By co-immunoprecipitation, pulldown and immunofluorescence co-localization assays, we revealed that STX2 selectively interacted with p85, a subunit of PI3K, and directly recruited p85 to the cytomembrane, thereby activating the AKT signaling pathway. We further demonstrated that the AKT activation was abolished by the use of a PI3K inhibitor (LY294002), which negatively affected STX2-mediated functions on trophoblasts.

**Conclusion:**

All together, our findings point to a crucial role for STX2 in PE progression. Our new insights also suggest that STX2 may be a potential diagnostic tool and a novel therapeutic target for treating PE.

## Introduction

Preeclampsia (PE) is a pregnancy complication characterized by high blood pressure, excess protein excretion in the urine and multiple organ dysfunction. With an incidence rate of 3–5% among pregnancies, PE accounts for a substantial number of maternal and neonatal deaths, and premature birth (Ukah et al., 2018). Currently, the usual therapy for PE is to give birth, but in certain cases this might not be possible given lack of fetal maturity (Esteve-Valverde et al., 2018). Although some factors have been shown to facilitate PE, including aspects of trophoblast dysfunction, such as the inadequate remodeling of the helicine artery, and immune dysfunction and excessive cell apoptosis, the exact pathogenesis and etiology of PE have remained largely unexplored (Sircar et al., 2015). Therefore, there is a pressing neeed for understanding the specific molecular mechanisms underlying PE, with the aim to find better therapies for this condition and also better strategies for preventing PE.

Syntaxin2 (STX2, also known as epimorphin) is an important member of the syntaxin family (Chen et al., 2009). The C-terminal domain of STX2 can bind to the cytomembrane, and STX2 acts as a key epithelial morphoregulator via its N-terminal domain (Miura et al., 2007). STX2 is reported to be involved in the morphogenesis and activation of epithelial cells in multiple tissues (Hirai et al., 1992, 1998), such as pancreatic ducts (Tulachan et al., 2006), the mammary gland (Hirai et al., 1998), the lung (Koshida and Hirai, 1997), and the intestinal epithelium (Fritsch et al., 2002). STX2 is also involved in the progression and metastasis of various cancers, such as mammary adenocarcinoma (Bascom et al., 2005), hepatocellular carcinoma (Jia et al., 2011), ovarian cancer (Yew et al., 2013), and colorectal cancer (Wang et al., 2018). Overall, these findings suggest a role for STX2 in cell proliferation, invasion and metastasis. However, the specific biological functions of STX2 and associated molecular mechanisms underlying the development of PE remain largely unknown.

A number of studies have shown a role for the AKT pathway preeclampsia pathogenesis (Wang et al., 2019; Chen et al., 2020), including its association with malformations in the developing placental labyrinth (Laguë et al., 2010; Tong et al., 2016). Downregulation of p-AKT in the extravillous trophoblast cells impacts the growth and invasion of these cells (Xu Y. et al., 2019). In addition, STX2 displayed anti-oxidative function via the activation of PI3K/AKT signaling pathways in intestinal epithelial cells (Iizuka et al., 2007).

The aim of this study was to examine the expression of STX2 protein in the placentas of women with PE. Our results indicate that STX2 protein expression was lower in the placenta of women with PE than in the placenta of women without PE. Using a combination of loss and gain of function experiments, we suggest a role for STX2 in promoting trophoblast growth and invasion potentially via the membrane recruitment of p85, a regulatory subunit of PI3K, which could lead to AKT activation in women with PE. Based on our findings, we propose that STX2 constitutes a novel biomarker that could be helpful in the early diagnosis of PE as well as a potential therapeutic target in this pregnancy complication.

## Materials and Methods

### Patients and Samples

Normal placentas (*n* = 35) were obtained from full-term births after cesarean section. Age-matched placentas were obtained from women severe preeclampsia (*n* = 40) after cesarean section. All placentas involved in this study were collected by procedures of planned cesarean section without aid of artificial labor. Placental tissues were obtained by a certified doctor by making a vertical incision across a normal area at the center, involving fetal and maternal placental surfaces. Tissues having calcified deposits or clots were excluded. The experiments were approved by the Ethics Committee of the Affiliated Hospital of Qingdao University, China. All volunteers participating in this study signed a written informed consent.

### Cell Culture

HTR-8/SVneo cells were acquired from the China Center for Type Culture Collection and kept in culture for all experiments. 10% (vol/vol) FBS/DMEM F12 was utilized for culturing the trophoblast line in an incubator at a temperature of 37°C at 5% CO_2_.

### Isolation and Culture of Primary Human Trophoblast Cells

Term placentas were collected from uncomplicated pregnancies after cesarean delivery. Isolation and culture of primary human trophoblast cells was done according to conventional methods. Specifically, we used a protocol based on the classic trypsin digestion and Percoll gradient centrifugation method, as previously described (Kliman et al., 1986; Sagrillo-Fagundes et al., 2016). In brief, the placental tissue was washed, sheared, and weighed, and digested in a solution containing trypsin and DNAse. The supernatant was collected and the pellet was kept. This process was performed in order to discard the outer syncytium and to keep the underlying trophoblasts. The pellet was then purified by centrifugation in a Percoll gradient, which allowed collection of the trophoblasts. At last, the trophoblasts were kept in culture using warm DMEM-HG (Thermo Fisher Scientific) with 10% vol/vol FBS.

### Transient Transfection and Lentivirus Infection

SiRNA transient transfections and lentivirus infection were performed with Lipofectamine 3000 (Invitrogen, California), as per manufacturer’s instruction. For cell transfection, lentiviruses containing either a non-targeting scrambled shRNA (Control) or a STX2-specific shRNA (sh-STX2) were used in gene-knockdown experiments (Hanbio, Shanghai, China). Control lentivirus (Vector) and lentiviral constructs expressing full-length STX2 (STX2) (Hanbio, Shanghai, China) were used for overexpression experiments. Green fluorescent protein (GFP) (Hanbio, Shanghai, China) was used for the selection of stable transfection.

In order to establish cell lines with consistent silencing or overexpression of STX2, we isolated single clones after puromycin treatment of the transfected cells.

The shRNAs sequences used were as follows: Control, 5′-GCAAGCTGACCCTGAAGTT-3′; sh-STX2, 5′-GCTTGAAG ATCTGAACAAA-3′.

### qRT-PCR Experiments (Quantitative Real-Time Polymerase Chain Reaction)

Total RNA was extracted with a Trizol reagent (Takara, Japan). Complementary DNAs were synthesized by using commercial reverse transcription kits (Invitrogen). qRT-PCR was performed with a SYBR Premix Ex Taq kit (Takara, Japan) and an ABI 7500 Sequencing Detection System, with β-Tubulin acting as a control.

The following primer sequences were used: STX2, forward, 5′-GCTCGGGACAGGCTTGAG-3′, reverse, 5′-GTCTGTGGTG GTTCTCCCAG-3′; β-Tubulin forward, 5′-TCCGAGTACCAGC AGTACCA-3′, reverse, 5′-ACAGAGGCAAAACTGAGCAC-3′.

### Western Blot

Cell lysis was conducted on ice with RIPA buffer (Sigma-Aldrich, St. Louis, United States). Cell lysates were centrifuged at 12,000 × g for 20 min and treated with 5x protein loading buffer. The membrane protein was isolated using the Minute^TM^ Plasma Membrane Protein Isolation Kit (SM-005, Invent Biotechnologies). Protein aggregates were then separated by SDS-PAGE and electro-transferred onto a PVDF membrane (Bio-Rad, Hercules, United States). Membranes were blocked in 5% (wt/vol) instant skim milk for 1 h at room temperature. Membranes were incubated at 4°C overnight with primary antibodies (SXT2 (ab12369, Abcam, Cambridge, MA; 1:1,000), p-AKT (ser473, #4060, CST; 1:1,000), AKT (#4685, CST; 1:1,000), p-GSK3β (ser9, #5558, CST; 1:1,000), β-catenin (#8480, CST; 1:1,000), β-Tubulin (#15115, CST; 1:5,000), PI3K p85 (#4257, CST; 1:1,000), PI3K p110α (#4249, CST; 1:1,000), PI3K p110β (#3011, CST; 1:1,000).

Following incubation with the primary antibody, membranes were then washed and incubated with secondary antibodies (1: 1,000; CST, Danvers, United States). Protein-antibody complexes were detected and quantified with a chemiluminescence detection system (Bio-Rad, United States?).

### Immunohistochemistry

Immunohistochemical analysis of placental tissues was performed on 4-μm paraffin sections. After dewaxing and rehydration, sections were treated with citric acid for antigen retrieval and blocked in 10% (wt/vol) Bovine Serum Albumin (BSA). Incubation with the primary antibody (polyclonal anti-SXT2 (ab12369, Abcam, Cambridge, MA; 1:500) was performed overnight at 4°C in a humid chamber. Slides were then washed with PBS and incubated with a secondary antibody (1:500; biotinylated secondary antibody; ZSGB-BIO) for 20 min at room temperature. Slides were then washed again in PBS and chromogenic detection was performed using a DAB (diaminobenzidine tetrahydro chloride) reagent kit was for 2–5 min, with hematoxylin used as counterstain. Imaging was performed with a Leica DM4000B microscope (Germany). Protein expression levels was performed with Image-Pro Plus 5.1 soft by two independent investigators blinded to the subgroups.

### Immunofluorescence

STX2-specific shRNA treated trophoblasts (HTR-8 or primary human trophoblast cells) were cultured for 24 h. To permeabilize cells for immunofluorescence assays, incubation with 0.5% (vol/vol) TritonX-100 for 15 min at room temperature was performed. Cells were then blocked with 6% (wt/vol) goat serum, and incubated with anti- PI3K p85 (ab86714, Abcam; 1:100) overnight at 4°C. Cells were then washed and incubated with a fluorescence-labeled second antibody (anti-488; AS001, abclonal; 1:300) for 1 h at room temperature. Then, cells were incubated with the anti-SXT2 antibody (ab12369, Abcam; 1:100) for 1.5 h and with another fluorescence-labeled second antibody (anti-CY3; AS007, abclonal; 1:300) for 1 h. Cells were washed once again and stained with DAPI (Guangzhou RiboBio, China; 1 μg/mL) for 5 min at room temperature. Imaging was performed on a confocal fluorescence microscope.

### CCK-8 Analysis

Cells (5 × 10^3^/well) were seeded in 96-well plates. Cell proliferation was monitored every day using a CCK-8 reagent (Thermo Fisher Scientific, Massachusetts, United States). Briefly, CCK-8 reagent was added to each well and cells were kept in culture for an extra period of 1.5 h. Colorimetric assays were then performed by measuring optical densities (OD) at 450 nm for each well using a microplate reader. Three independent experiments were performed to determine the growth curves.

### EdU Assay

The Click-iT^®^ EdU Alexa Fluor^®^ 488 Cell Proliferation Assay Kit (Molecular Probes, Invitrogen, OR, United States) was used to perform the EdU assay according to manufacturer’s instructions. Briefly, cells were treated prior to pulse-labeling with 10 μM and 0.1 μM EdU for 3 h.

Harvested cells were washed twice with 1% BSA. Following incubation in Click-iT^®^ fixative for 15 min in the dark, cells were washed in a saponin-based permeabilization and washing reagent (1×). Then, the cells were incubated in 1 × Click-iT^®^ EdU reaction cocktail for 30 min in the dark and washed again. Finally, cellular DNA was stained with a DNA staining solution prepared according to manufacturer’s instructions. Samples were analyzed with a BD FACSCalibur^TM^ flow cytometer (BD Biosciences, San Jose, CA).

### Ki67 Cell Proliferation Assay

Harvested cells (about 1 × 10^6^) were incubated with an anti Ki67-FITC monoclonal antibody (clone MIB-1, DAKO, Glostrup, DK) following use of Leucoperm reagents A and B (Serotec) and lysing solution dissolution treatment (BD lysing solution, Becton Dickinson, San Josè, CA, United States). Then, samples were resuspended, washed and immediately analyzed on a BD Accuri C6 flow cytometer (Becton Dickinson). A minimum of 10,000 cells were acquired both for immunophenotype and Ki67 determination. The CFlow Plus software (Becton Dickinson) was used to analyze the data. Cell proliferative activity was based on the percentage of Ki67 positive cells (KI67I). Results were shown in the form of a scattergram gate of FSC vs. SSC, and the percentage of positive cells was calculated based on SSC vs. fluorescence intensity values.

### Transwell Assay

Treated cells (5 × 10^4^/well, 100 μL) without FBS were seeded in the upper chamber of Transwell plates (Corning, NY, United States) with 8 μm membrane. Then, 750 μL complete culture medium with 10% (vol/vol) FBS was added to the lower chamber. For the invasion assay, the membrane was covered with 100 μL BD Matrigel^TM^ matrix at a 1:8 dilution. Then, cells were cultured for 16–24 h, fixed with 4% paraformaldehyde and dyed with a crystal violet solution. The number of penetrated cells on the membrane was considered a measure of the cell migration and invasion capability. Experiments were done in triplicate.

### Colony Forming Assay

To observe the colony formation condition of cells, about 1,000 HTR-8/SVneo or JEG-3 primary human trophoblast cells cells were seeded separately in six-well plates. After 14 days, colonies were fixed in paraformaldehyde solution and dyed with a crystal violet solution.

### Co-immunoprecipitation (Co-IP) and GST Pull-Down Assay

Cells were lysed in a lysis buffer (Pierce, Rockford, United States). Lysates were then centrifuged and the supernatant was cleared by incubation with Protein A/G magnetic beads (Thermo Fisher Scientific) at 4°C temperature for one hour. The pre-cleared supernatant was then immunoprecipitated by incubation with the primary antibody SXT2 (ab3265, Abcam; 1:200) overnight at 4°C. Protein complexes were then incubated with Protein A/G magnetic beads for 1 h at 4°C and analyzed by western blot.

The proteins tagged with GST and His were amplified in Escherichia coli BL. Following the instructions, GST-tagged proteins were incubated with glutathione-Sepharose 4B beads (Amersham Biosciences) for purification. His-tagged proteins were purified by nickel affinity resins (Merck). After adding 20 μm of glutathione-Sepharose 4B beads, the proteins tagged with His and GST or GST alone protein were mingled in PBS binding buffer (Takara’s PBS, pH 7.4, 4°C, 2 h) and then the mixture was cultured for 1 h with nutation. Finally, the beads were washed and eluted with 2x SDS sample buffer for western blot analysis.

### Statistical Analysis

Unpaired two-tail *t*-test was used to perform all statistical analyses. All data were reported as the mean ± standard error mean deviations from at least three independent experiments. GraphPad Prism version 7.00 software program (GraphPad; La Jolla, United States) was used to analyze the data. A *p*-value of less than 0.05 was considered statistically significant.

## Results

### STX2 Is Downregulated in the Placenta of Pregnant Women With PE

**FIGURE 1:**
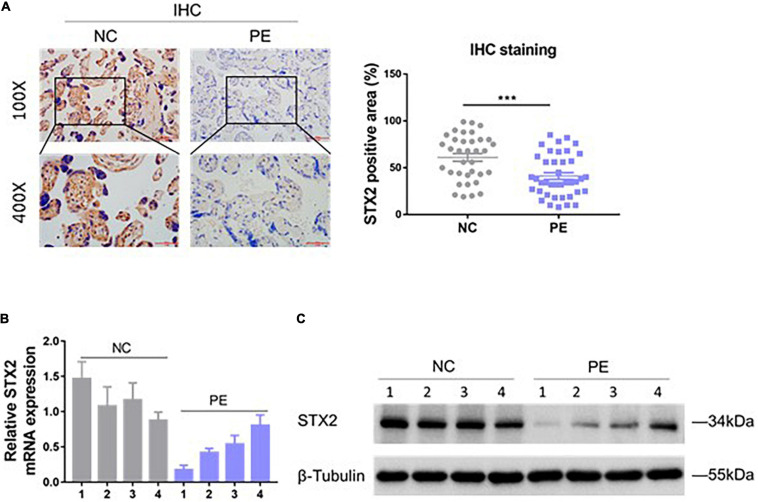
STX2 is downregulated in the placenta of PE pregnancies. **(A)** Representative images of IHC staining of STX2 in normal control (NC; *n* = 35) tissues and PE (*n* = 40) tissues. Scale bar: 100 μm (100×); 50 μm (400×). Student’s *t*-test: ***P < 0.001. **(B)** Relative STX2 mRNA levels of placenta tissues from pregnant women with PE or normal control pregnancy (NC) as detected by qRT-PCR (*n* = 4/group). **(C)** Relative STX2 protein levels of placenta tissues from pregnant women with PE or normal control pregnancy as detected by western blot (*n* = 4/group). β-Tubulin was used for normalization. All the experiments were repeated three times independently.

To investigate if STX2 protein expression was altered in the placenta of women with PE, we obtained placental tissues from 35 females with normal pregnancy and from 40 women with PE. Using immunostaining techniques, we observed that STX2 proteins were largely localized on the cellular membrane and cytoplasm of cytotrophoblasts (Figure 1A). Based on our analysis, STX2 protein had a lower expression level in the PE group than in the control (NC) group. Analysis of STX2 mRNA levels, by qRT-PCR, and of STX2 protein levels, by western blot, in placental tissues further confirmed the IHC results (Figures 1B,C). All together, these results demonstrate that STX2 expression downregulated significantly in the placenta of women with PE.

### STX2 Promoted Proliferation, Migration, and Invasion of Trophoblasts

**FIGURE 2:**
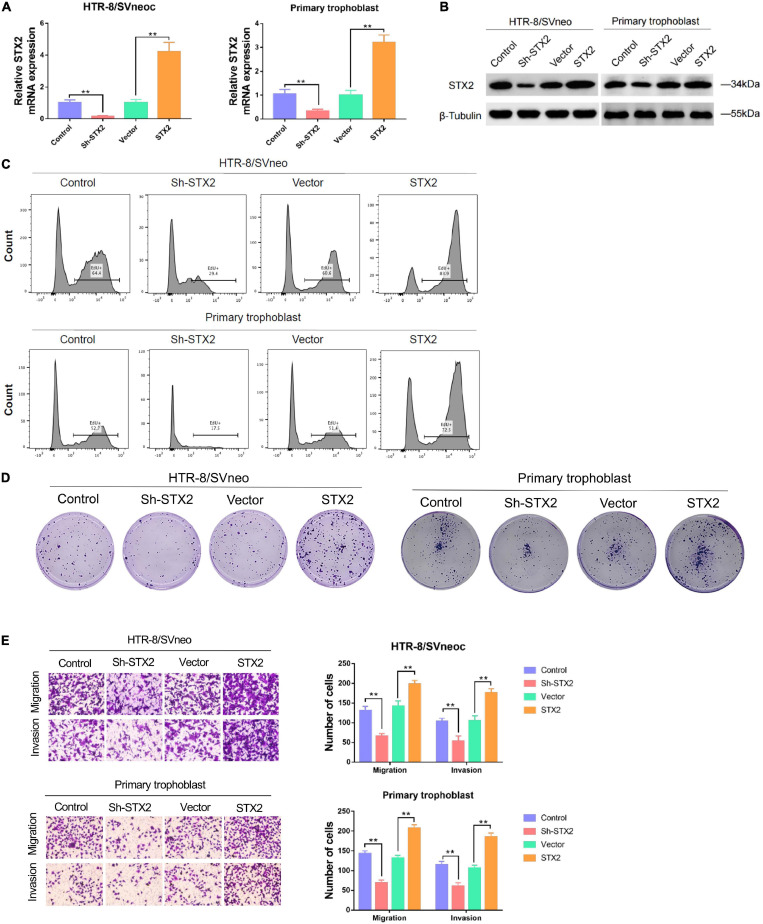
STX2 promotes proliferation, migration and invasion of trophoblast cells. **(A)** qRT-PCR analysis and **(B)** western blot analysis of STX2 expressions in HTR-8/SVneo and primary human trophoblast cells transfected with Sh-STX2 and STX2-expressing lentivirus. **(C)** EdU assay and **(D)** colony forming assay of control and experimental cells in which STX2 was knocked down or overexpressed. Down-regulated STX2 reduced cell proliferation, whereas up-regulated STX2 increased cell proliferation. **(E)** Transwell assays of control and experimental cells in which STX2 was knocked down or overexpressed. Down-regulated STX2 inhibited cell migration and invasion, whereas up-regulated STX2 promoted cell migration and invasion. All the experiments were repeated three times independently. Data are represented as the mean ± SEM. Student’s *t*-test: **P < 0.01.

To further explore the expression of STX2 in placental trophoblast cells, we used HTR-8/SVneo cell lines and primary human trophoblast cells for STX2 knockdown and overexpression experiments. Analysis of mRNA and protein levels was used to evaluate the efficacy of lentivirus transfection (Figures 2A,B). EdU assays and clone forming assays were used to determine the proliferation ability of trophoblast cells with different treatments. We observed that cell proliferation was reduced substantially in HTR-8/SVneo and primary human trophoblast cells with Sh-STX2 expression, but increased in these cells, compared with their control cells (Figures 2C,D). CCK-8 assays and Ki67 cell proliferation assays provided the similar results (Supplementary Figures 1A,B). Transwell assay suggested that STX2 knockdown significantly reduced cell migration and invasion, while STX2 overexpression promoted these behaviors (Figure 2E). These results suggest that STX2 may have a role in placental trophoblasts by regulating their proliferation, migration, and invasion capability.

### STX2 Activated AKT Signaling Pathway via Membrane Recruitment of PI3K p85

**FIGURE 3:**
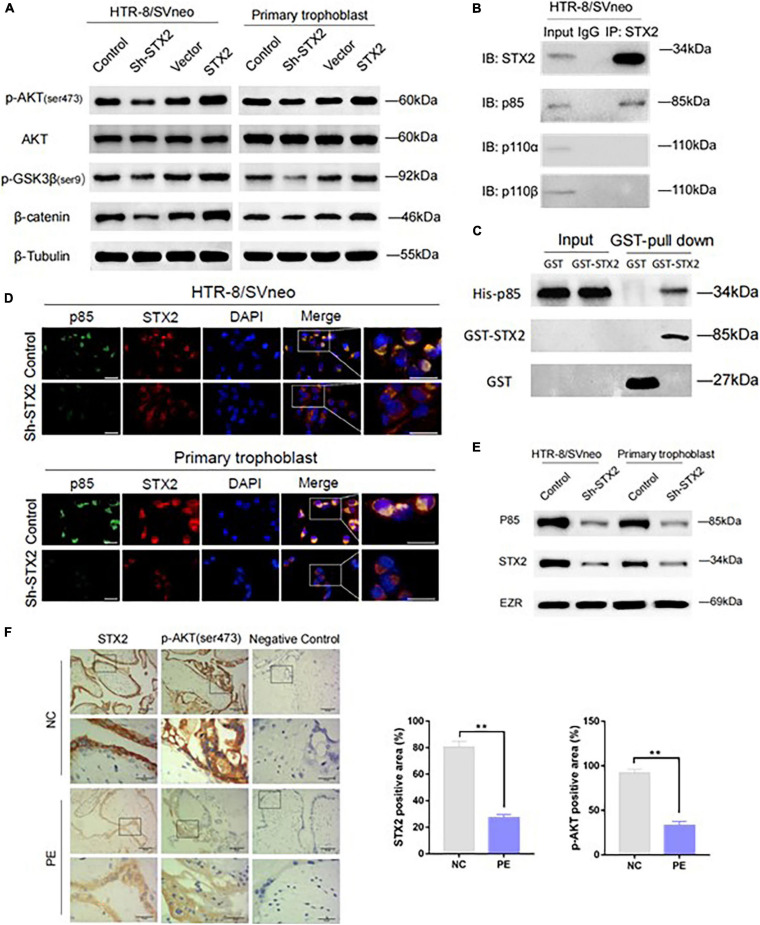
STX2 activates the AKT signaling pathway via membrane recruitment of PI3K p85. **(A)** Western blot analysis of p-AKT (ser473), AKT, p-GSK3β(ser9), and β-catenin in stably transfected HTR-8/SVneo and primary human trophoblast cells. β-Tubulin was used for normalization. **(B)** Co-IP results showing cell lysates were immunoprecipitated (IP) with antibodies for STX2, followed by western blot using PI3K p85, PI3K p110α, PI3K p110β, or STX2 antibodies. **(C)** GST pull-down assay was performed to clarify the binding between STX2 and PI3K p85 *in vitro*. **(D)** Confocal microscopy detection of subcellular distribution of STX2 (red) and PI3K p85 (green) in HTR-8/SVneo and primary human trophoblast cells transfected with Sh-STX2. Scale bar: 25μm. **(E)** Western blot analysis of the expression of p85 in membrane protein of control and experimental cells in which STX2 was knocked down. **(F)** Representative images of IHC staining for STX2 and p-AKT (ser473) in patients with PE (*n* = 4/group). Data are represented as the mean ± SEM. Student’s *t*-test: **P < 0.01. All the experiments were repeated three times independently.

AKT pathway plays a critical role in trophoblastic growth, migration and invasion (Zhu et al., 2016; Wang et al., 2019; Xu B. et al., 2019). STX2 has been shown to regulate several different signaling pathways or molecular factors, such as FAK/ERK (Jia et al., 2011), β-catenin (Yew et al., 2013), and NF-κB (Wang et al., 2018). Based on previous studies that investigate the cross-talk between STX2 and these classical signaling pathways, it is reasonable to speculate that STX2 may regulate AKT signaling pathway activation in PE. We examined activation of AKT in both HTR8/SVneo and primary human trophoblast cells. We observed that, in stably transfected cells, levels of phosphorylated AKT (Ser473), as well as its downstream genes, including phosphorylated GSK3β (ser9) and β-catenin (Katoh and Katoh, 2006; Song et al., 2018; Xu B. et al., 2019) changed according to changes in STX2 levels (Figure 3A).

As shown in Figure 1A, STX2 protein was found to be located on the cytomembrane and cytoplasm. It’s been shown that some membrane proteins can interact with PI3K and enhance phosphatidylinositol-3,4,5-trisphosphate (PIP3) production, thus activating the AKT signaling pathway (Zhang et al., 2013; Zhou et al., 2019). In addition, STX2 has been reported to interact with TRAF6 to activate the NF-κB pathway in colorectal cancer cell lines (Wang et al., 2018). Based on these findings, we wondered if STX2 was able to recruit PI3K directly to the cytomembrane to activate the AKT pathway in trophoblasts. Our co-immunoprecipitation results suggested that STX2 could be bound to p85, a regulatory subunit of PI3K, but exhibited little affinity with p110α and p110β, the catalytic subunits of PI3K (Figure 3B). GST pull-down assay further demonstrated that STX2 directly bound to p85 *in vitro* (Figure 3C). Immunofluorescence analysis further confirmed that STX2 and p85 proteins co-localized on the cytomembrane and cytoplasm of control group in HTR8/SVneo and primary trophoblast cells. In contrast, STX2 and p85 proteins were found to be scattered separately in the cytoplasm of cells in Sh-STX2 groups (Figure 3D). Then we extracted the membrane protein of the two cells, western blot showed that the protein expression of p85 was decreased significantly in the sh-STX2 groups (Figure 3E). We have also performed IHC staining of phosphorylated AKT (Ser473) in the placental tissues of women with PE previously described in Figure 1A. We observed that STX2 protein expression was positively correlated with p-AKT expression in a statistically significant manner (Figure 3F). Overall, these results suggest that STX2 may modulate AKT signaling pathway via recruitment of PI3K to the membrane.

### STX2 Promotes Trophoblast Proliferation, Migration, and Invasion via Activation of the AKT Pathway

**FIGURE 4:**
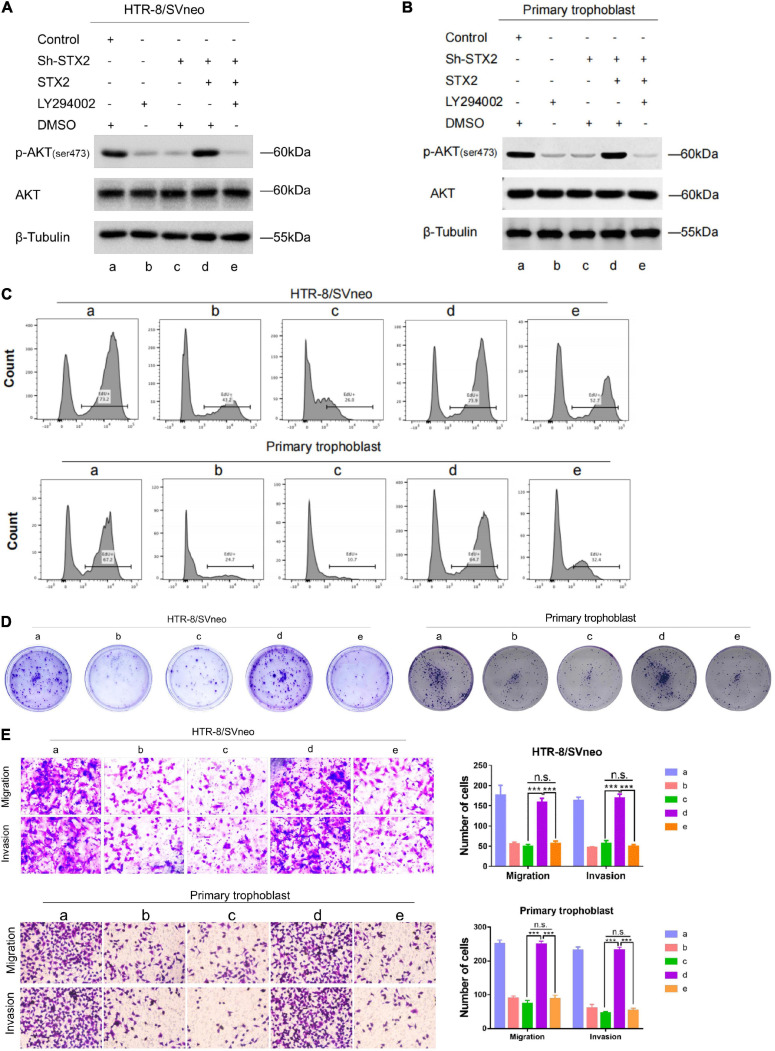
STX2 promotes trophoblasts proliferation, migration and invasion through activating PI3K-AKT pathway. **(A,B)** Western blot analysis of p-AKT (ser473), AKT in indicated stably transfected HTR-8/SVneo and primary human trophoblast cells treated with LY294002 (PI3K inhibitor, 10 μM) or DMSO (negative control) for 48 h. **(C)** EdU assay and **(D)** colony forming assay were used to evaluated the proliferation of control and experimental cells in which STX2 was stably transfected with or without LY294002 treatment. **(E)** Transwell assays were used to evaluated the migration and invasion of control and experimental cells in which STX2 was stably transfected with or without LY294002 treatment. All the experiments were repeated three times independently. Data are represented as the mean ± SEM. Student’s *t*-test: ***P < 0.01.

To investigate the role of AKT in STX2-induced trophoblast proliferation, migration and invasion, cells were treated with an inhibitor of PI3K (LY294002). We transfected the above two cells with a sh-STX2-expressing lentivirus construct, and thus reversed the expression of STX2 (Figure 4A). Then, all stably transfected cells were treated either with LY294002 or DMSO for 48 h. Interestingly, after excluding off-target effects of the shRNA overexpression, we observed that STX2 effectively reversed the activation of the AKT pathway. However, use of the PI3K inhibitor completely abolished the rescue or overexpression effects of STX2 on the AKT pathway (Figures 4A,B). We investigated cell proliferation by EdU and colony formation assays (Figures 4C,D), and observed that PI3K inhibition restricted the trophoblast cells proliferation. Viability of the two cells was also markedly reduced after PI3K inhibition treatment (group c vs. d, d vs. e, *P* < 0.01; c vs. e, *P* > 0.05; Supplementary Figure 1C) by CCK-8 analysis. Cell migration and invasion were also affected in a manner consistent with the findings described above assessed by the transwell assays (Figure 4E).

**FIGURE 5:**
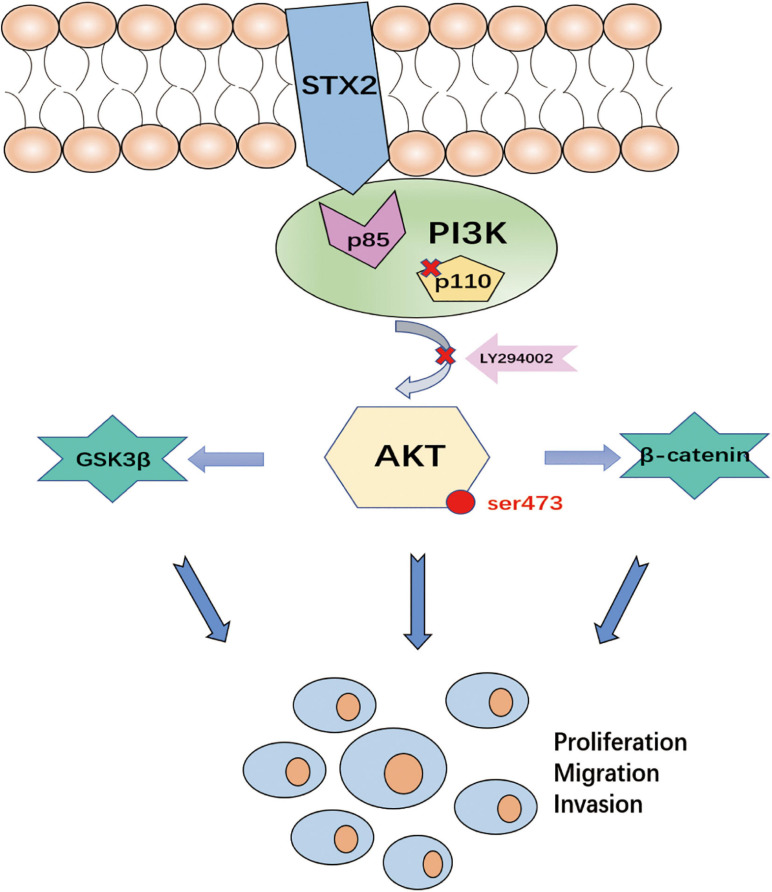
The diagrammatic presentation of the underlying mechanism of STX2 promoting PE development. STX2 activated the PI3K-AKT pathway by interacting with PI3K p85 to prompt the trophoblast proliferation, migration and invasion. Therefore, down-regulation of STX2 in placenta contributed to the development of PE.

In conclusion, our results suggest that the AKT pathway plays a vital role in STX2-induced trophoblast proliferation, migration and invasion (Figure 5).

## Discussion

Earlier studies revealed a correlation between STX2 expression cell proliferation, invasion and metastasis in various diseases. For example, STX2 has been shown to play an essential in human hepatocellular carcinoma invasion and metastasis (Jia et al., 2011). In this study, our results from IHC staining, qRT-PCR and western blot analysis demonstrate downregulation of both STX2 mRNA and protein in placental tissues of women with PE. Through loss or gain of function experiments, we showed that STX2 depletion attenuated cell proliferation, invasion, and migration, while overexpression of STX2 increased cell proliferation, invasion, and migration in both HTR-8/SV neo and primary human trophoblast cells. In addition, we showed that the re-expression level of STX2 in HTR-8/SVneo-Sh-STX2 cells reached that of controls (or in primary human trophoblast cells), further suggesting a modulating effect of STX2 on trophoblasts function (Figures 4A,B).

Given that knockdown and overexpression of STX2 may play an important role in the progression of PE, investigations into the mechanisms underlying these effects are of utmost importance. Multiple signaling pathways have been involved in regulating cellular proliferation and invasion. For example, the PI3K/AKT pathway has been shown to be vital for numerous aspects of cellular activities both in physiological and pathological conditions. Several studies have shown that certain cell behaviors, including proliferation, migration and invasion, could be subdued by inhibiting and targeting upstream molecules in the PI3K/AKT signaling pathway in PE (Zhu et al., 2016; Wang et al., 2019; Xu Y. et al., 2019). Blocking the PI3K/AKT signaling pathway also reduced the expression of sFlt1 in placentas of women with PE (Park et al., 2010). Moreover, desensitization of the PI3K/AKT pathway, which accounted for endothelial dysfunction, was also observed in PE and increased the level of soluble endoglin, an antagonist of TGF-β signaling (Cudmore et al., 2012). In this study, we found that STX2 expression was correlated with the level of p-AKT, as well as of its downstream genes, p-GSK3β and β-catenin. We hypothesize that the role of STX2 in the regulation of the AKT pathway might be key in trophoblast growth and invasion. Further investigations on this topic will help unveil the possible mechanisms underlying the effect of STX2 on the growth and invasion capabilities of these cells. Indeed, a fundamental question that remains unanswered is how STX2 promotes trophoblast proliferation and invasion. It has been known that membrane-residing PI3K phosphorylates phosphatidylinositol-4,5-bisphosphate (PIP2) into PIP3, which in turn recruits and activates corresponding effectors, such as PDK1 and AKT (Balla et al., 2009; Miao et al., 2010; Huang et al., 2011) in (please mention which cells/tissues). Certain membrane proteins have been shown to bind PI3K, enhance PIP3 production and activate AKT signaling (Carnero et al., 2008). Our results from co-immunoprecipitation experiments suggested that STX2 formed a high affinity complex with p85, a subunit of PI3K, but not with p110α/β. Immunofluorescence analysis also suggested that most of p85 protein co-localized with STX2, and that STX2 and p85 co-localization was reduced following STX2 knockdown. An interaction between STX2 and p85 is possible, and in agreement with a recent study that showed an interaction between STX2 and another cell surface protein, TRAF6 (Wang et al., 2018). However, further studies are needed to determine if STX2 and PI3K interact with each other directly or indirectly.

Moreover, LY294002, currently the most widely used PI3K inhibitor (Wang et al., 2019; Xu Y. et al., 2019), can effectively block proliferation, migration and invasion of trophoblasts induced by STX2 rescue or overexpression, confirming the important role of AKT signaling activation regulated by STX2 in the development of PE. These findings indicate that STX2 may activate the AKT signaling pathway by direct binding to and recruiting PI3K to the cytomembrane, and thus provide our deeper understanding of the role of STX2 in regulating PI3K/AKT signaling in trophoblasts. Our results showed a relationship between STX2 and PI3K pathway in pathogenesis of PE, which gave new mechanistic insight and made possible the early diagnosis of PE. However, there is still a long way to go before the PI3K pathway inhibitor could become a treatment in PE.

In summary, our study demonstrated that STX2 is downregulated in placental tissues of women with PE, possibly resulting in reduction of trophoblast proliferation and invasion. Our results also suggest that STX2-mediated cellular dysfunction of trophoblasts is induced by activation of the AKT signaling through membrane recruitment of p85. Naturally, further studies are still needed to clarify the specific mechanism of STX2-induced PE, meanwhile the interaction of STX2 and PI3K is an exploratory orientation to reveal the pathogenesis of PE.

## Data Availability Statement

The raw data supporting the conclusions of this article will be made available by the authors, without undue reservation.

## Ethics Statement

The studies involving human participants were reviewed and approved by the Ethics Committee of Affiliated Hospital of Qingdao University. The patients/participants provided their written informed consent to participate in this study.

## Author Contributions

Y-HW conceived and designed the study and revised the article critically. YL, X-LS, and C-LM performed the experiment. YL drafted the manuscript. ChL and YZ analyzed and interpreted the data. W-TL and CaL were responsible for reagents and materials. All authors contributed to the article and approved the submitted version.

## Conflict of Interest

The authors declare that the research was conducted in the absence of any commercial or financial relationships that could be construed as a potential conflict of interest.

## Supplementary Material

Supplementary Figure 1 CCK-8 and Ki67 assays tested the trophoblast proliferation property in different treated groups. **(A)** CCK-8 assay of control and experimental cells in which STX2 was knocked down or overexpressed. **(B)** Ki67 cell proliferation assay of control and experimental cells in which STX2 was knocked down or overexpressed. **(C)** CCK-8 assay was used to evaluated the proliferation of control and experimental cells in which STX2 was stably transfected with or without LY294002 treatment. All the experiments were repeated three times independently.
