# Supplementary figures and images for: STX2 Promotes Trophoblast Growth, Migration, and Invasion Through Activation of the PI3K-AKT Pathway in Preeclampsia
Source: Front Cell Dev Biol. 2021 Jul 6;9:615973. doi: 10.3389/fcell.2021.615973 (PMC8292021; doi:10.3389/fcell.2021.615973)

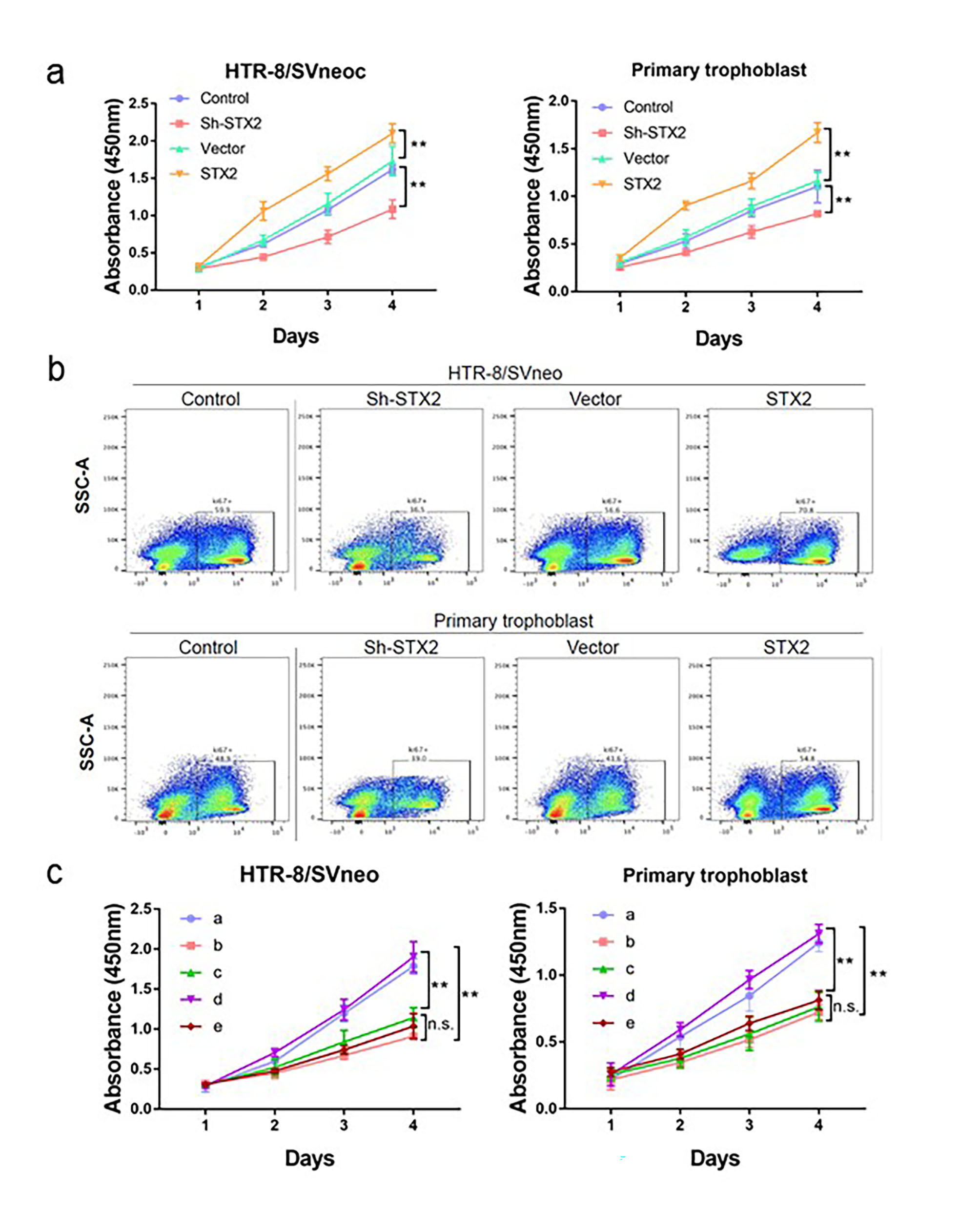

Supplement: Supplementary Figure 1 — CCK-8 and Ki67 assays tested the trophoblast proliferation property in different treated groups. (A) CCK-8 assay of control and experimental cells in which STX2 was knocked down or overexpressed. (B) Ki67 cell proliferation assay of control and experimental cells in which STX2 was knocked down or overexpressed. (C) CCK-8 assay was used to evaluated the proliferation of control and experimental cells in which STX2 was stably transfected with or without LY294002 treatment. All the experiments were repeated three times independently. [file Image_1.JPEG]
